# The role and prognostic value of apoptosis in colorectal carcinoma

**DOI:** 10.1186/1472-6890-13-24

**Published:** 2013-10-10

**Authors:** Julia Alcaide, Rafael Funez, Antonio Rueda, Elisabeth Perez-Ruiz, Teresa Pereda, Isabel Rodrigo, Rafael Coveñas, Miguel Muñoz, Maximino Redondo

**Affiliations:** 1 Division of Medical Oncology, Onco-Hematology Department, A.S. Costa del Sol, Autovia A-7, Km 187, Marbella, Malaga, CP 29603, Spain; 2 Department of Pathology, A.S. Costa del Sol, Autovia A-7, Km 187, Marbella, Malaga, CP 29603, Spain; 3 Red de Investigacion en Servicios de Salud (REDISSEC), Spain; 4 Research Laboratory, Hospital Infantil Universitario Virgen del Rocio, Sevilla, CP 41013, Spain; 5 Research Unit, A.S. Costa del Sol, University of Malaga, Marbella, Malaga, CP 29603, Spain

**Keywords:** Apoptosis, Colorectal carcinoma, Prognosis

## Abstract

**Background:**

Alterations to apoptosis are a common occurrence in human tumours. The aim of our study was to determine the influence of apoptotic variations on the carcinogenesis and prognosis of colorectal carcinomas (CRCs).

**Methods:**

A TUNEL assay was performed on archival material from 103 colorectal carcinomas, 26 adenomas and 20 samples of normal epithelia.

**Results:**

The number of apoptotic cells was higher in CRCs (1.09 ± 0.13) than in adenomas (0.38 ± 0.23, p = 0.059) and normal epithelium (0.06 ± 0.04, p = 0.001). In addition, the apoptotic index (AI) was greater in metastatic disease (stage IV) than in other stages (p = 0.017). No relationship was found between apoptotic rates and age, gender or tumour grade. However, patients with tumours that showed higher AI values had a significantly lower disease-free survival (DFS) and overall survival (OS) than those with tumours that had lower AIs (p = 0.020 and p = 0.027). In a multivariate Cox proportional hazards model, AI remained a significant independent predictor of survival.

**Conclusions:**

We conclude that disregulated apoptosis is an important event during CRC development and progression. Higher AIs are associated with more aggressive tumours and a poorer prognosis for patients with CRC.

## Background

Currently, colorectal cancer (CRC) represents an important public health problem due to its high incidence and mortality. It is the third-most-common tumour type, and approximately one million new cases of CRC are diagnosed per year worldwide [1]. The CRC survival rates are primarily determined by the stage of the tumour at diagnosis, as determined by the TNM (Tumour-Node-Metastases) classification system. At 5 years, 90% of patients with a localised tumour (a tumour that is confined to the intestinal wall) will be alive, whereas this percentage decreases to 60-70% if the tumour has spread to regional lymph nodes and is only approximately 5-10% for cases of CRC that involve metastatic disease. Moreover, approximately 40-50% of the patients that initially present with early stages of CRC will relapse. Despite recent improvements in CRC management, there remains a need to find biomarkers that provide prognostic information and guide therapy decisions.

Most CRCs progress through a multistep process that involves a series of genetic alterations; these alterations produce a phenotypic progression from normal tissue to adenoma to carcinoma. This tumourigenesis sequence is proposed by the Vogelstein model and accounts for approximately 85% of all CRCs [2]. According to this model, adenomas of the colorectum are precursor lesions that may undergo malignant transformations and develop into adenocarcinomas over a period of months or years. This development involves three physiological phenomena: proliferation, differentiation and cell death. It has been demonstrated that an increase in proliferative activity occurs concurrently with the worsening of dysplasia during the adenoma-carcinoma transition. However, the role of apoptosis in this process has not yet been completely clarified.

Apoptosis may occur via two major interconnected pathways: the extrinsic or death receptor-mediated pathway, which is activated by the binding of specific ligands (such as FasL, TNF-α and TRAIL) to the receptors of cell surfaces; and the intrinsic or mitochondrial-mediated pathway, which is regulated through proteins of the Bcl-2 family and triggered either by the loss of growth factor signals or in response to genotoxic stress. Therefore the replication of cells with DNA damage is generally avoided because harmful genomic alterations typically induce the activation of apoptosis. It has been widely accepted that alterations in the physiologic response to DNA damage can facilitate the accumulation of oncogenic mutations; this accumulation may eventually lead to the development of neoplasia.

If the mechanisms that are necessary for maintaining the balance between proliferation and apoptosis function properly, then the homeostasis of the colonic epithelium in the intestinal crypt will be maintained. However, in this system, which involves a very high cell turnover rate, the down-regulation of apoptotic function would allow uncontrolled cell proliferation and tumour development. In fact, in several studies, a progressive inhibition of apoptosis during the mutation of cells from normal mucosa to CRC has been demonstrated [3,4]. However, other studies have suggested a trend towards increased apoptotic index (AI) during the process of CRC development [5-8]. Therefore, further studies are needed to confirm this trend. In addition, given the emergent evidence indicating the relevance of apoptosis to the pathogenesis and progression of CRC, the potential prognostic implications of apoptotic rates have become increasingly intriguing. Nevertheless, there is a paucity of works demonstrating the prognostic significance of apoptosis in CRC, and only some researches have reported statistically significant worse outcomes for patients with higher AIs [9,10]. In this study, we investigated whether differences in apoptotic rates could be related to carcinogenesis and to the survival of CRC, and we report the first evidence that high AI is associated with a significant decrease not only in overall survival (OS), but also in disease-free survival (DFS) among patients with CRC.

## Methods

### Patients and tissue samples

After excluding patients that had previously been treated with chemotherapy and/or radiation therapy, a total of 103 CRC from patients who had experienced tumour resection at the Costa del Sol Hospital between January 2006 and December 2007 were studied. As well 26 samples belonging to patients with adenomas and 20 normal colon tissues were studied. Sections of normal colonic mucosa were obtained from surgical specimens, not adjacent but remote from carcinoma and considered as normal by pathologists. All of the tissue samples were routinely fixed in 10% buffered formalin and embedded in paraffin blocks. On the other hand, the areas of tumours for the TUNEL assay were selected as well by expert pathologists, excluding necrotic areas. Table 1 lists the characteristics of the 103 patients with CRC of this study. The clinical data were obtained from the tumour registry and hospital charts of the Costa del Sol Hospital, and the present study was approved by the Research Ethics Committee of that hospital. Specimens were examined from 57 men and 46 women. The mean age of the patients at surgery was 70 years (range: 45–91). The follow-up time was calculated to be from the time of the initial pathologic diagnosis to the last date of contact with the patient, and the median observation period was 50 months (range: 12–96). The CRCs were characterised by grade and stage in accordance with the WHO and TNM classification systems.

**Table 1:** Patients characteristics (n = 103)

**Parameter**	**Patients (n)**	**%**
Gender		
Male	57	55
Female	46	45
Age (years)		
Median	70	
Range	45 - 91	
Grade		
I	16	16
II	56	54
III	31	30
Location		
Cecum and ascending colon	22	21
Transverse colon	11	11
Sigmoid and descending colon	39	38
Rectum	31	30
Stage		
I	16	15
II	49	48
III	24	23
IV	14	14
Recurrence/Progression		
No	57	55
Yes	46	45

### In situ localisation of apoptotic cells (TUNEL assay)

To detect apoptotic cells, the in situ labelling of the 3’-ends of the DNA fragments that were generated by apoptosis-associated endonucleases was performed using a commercial apoptosis detection kit (Roche Diagnostics GmbH, Mannheim, Germany). Briefly, deparaffinised sections were incubated with 20 mg/ml of proteinase K (Sigma Chemical Co., St. Louis, MO, USA) for 15 minutes. Following a rinse in PBS, the slides were covered with a terminal deoxynucleotidyl transferase and nucleotide mixture at a 1:35 dilution for 60 minutes at 37°C. The slides were then covered with an antifluorescein antibody that was conjugated with alkaline phosphatase. After the substrate reaction had occurred, the stained cells were analysed under a light microscope. The pretreatment of sections with DNase served as a positive control for the enzymatic procedures; for a negative control, the same procedures were performed without the inclusion of the enzyme. The established morphological features that were used to identify apoptosis on H&E slides were also used for TUNEL-stained slides. Cells were defined as apoptotic if the whole nuclear area of the cell was positively labelled. Apoptotic bodies were defined as small, positively labelled globular bodies in the cytoplasm of the tumour cells; these bodies were found either singly or in groups. In total, 1000 cells were counted for each specimen. The number of positively stained cells was then divided by 1000 to estimate the percentage of apoptotic cells in each specimen. We used the median of the apoptotic percentage in our samples (1%) (range: 0-6%) as a threshold value for survival studies. The evaluation of the specimens was independently performed by two investigators, who had no access to either the clinical data or the laboratory information for the patients of the study.

### Statistical analysis

Statistical analyses were performed using the SPSS statistical software program (SPSS Inc., Chicago, IL, USA). The analysis of the association between apoptosis and various prognostic factors for CRC used the X^2^ test for categorical variables and the analysis of variance test for continuous variables (which were natural log transformed if necessary). The relapse-free survival was estimated using the Kaplan-Meier method, and the survival curves were compared with the log rank test. Cox’s proportional hazards survival analysis was used to determine the relative risk through multivariate analyses. The p <0.05 level of statistical significance was employed throughout this study.

## Results

### Apoptosis

**Figure 1:**
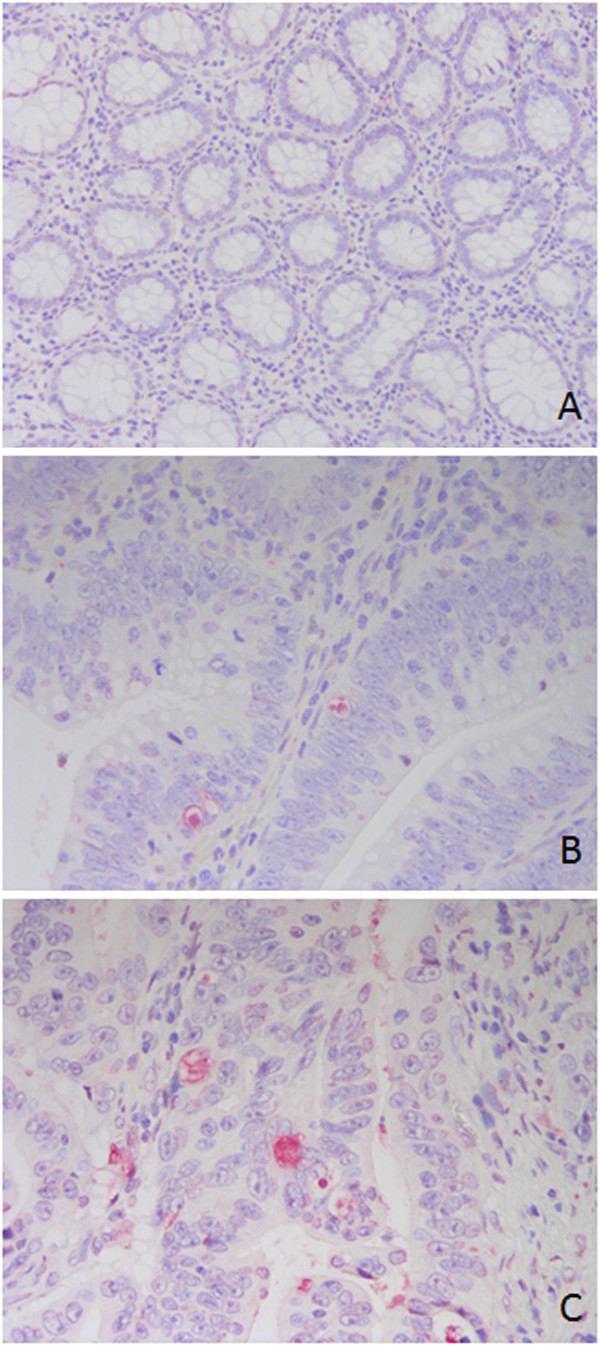
**Apoptosis detected by TUNEL assay.** Positively stained cells were very scarce in normal mucosa **(A)**, increased in adenomatous polyps **(B)** and especially in adenocarcinomas of the colon **(C)**. Magnification, 200x **(A)**, 400x **(B, C)**.

A TUNEL assay was performed to quantify apoptosis in colon tissue and determine whether the frequency of apoptosis was related to tumourigenesis. The apoptotic rate of cells was greater in CRCs (mean 1.09 ± 0.13) than in adenomas (mean 0.38 ± 0.23, p = 0.059), and the apoptotic rate was significantly higher in CRCs than in normal epithelial cells (mean 0.06 ± 0.04, p = 0.001) (Figure 1). Therefore, apoptosis is related to neoplastic transformation. With respect to the tumour stage, we found that the AIs were much greater in metastatic disease cases (stage IV) than in the localised stages of the disease (stages I, II and III), with p = 0.017. Apoptotic rates did not correlate with the gender, age or tumour grade of patients.

### Disease-free survival (DFS)

**Figure 2:**
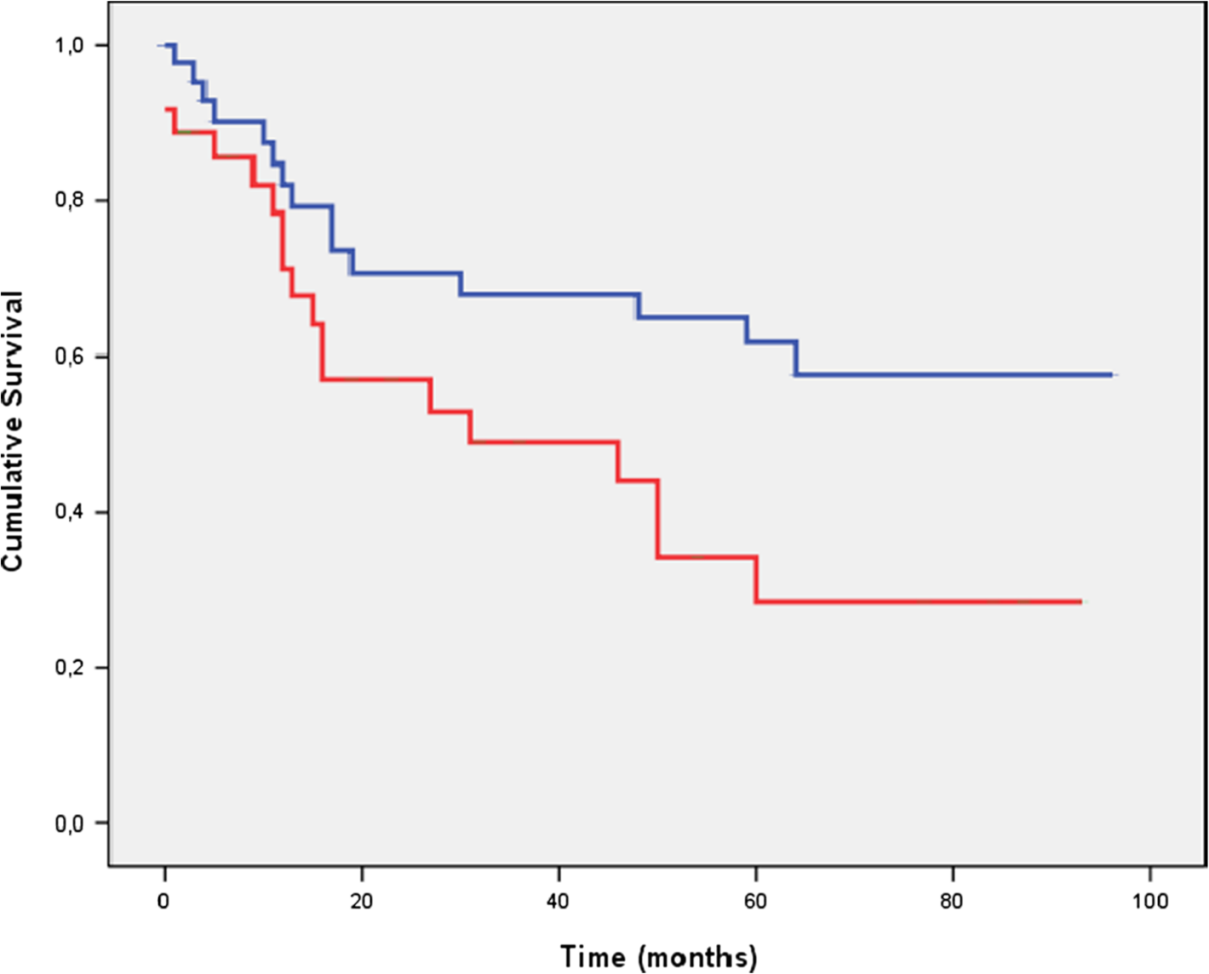
**The disease-free survival (DFS) of patients, grouped by apoptotic cell rates.** The survival of patients with primary tumours presenting high AIs (lower line) is significantly shorter than the survival of patients presenting tumours with low AIs (upper line).

**Figure 3:**
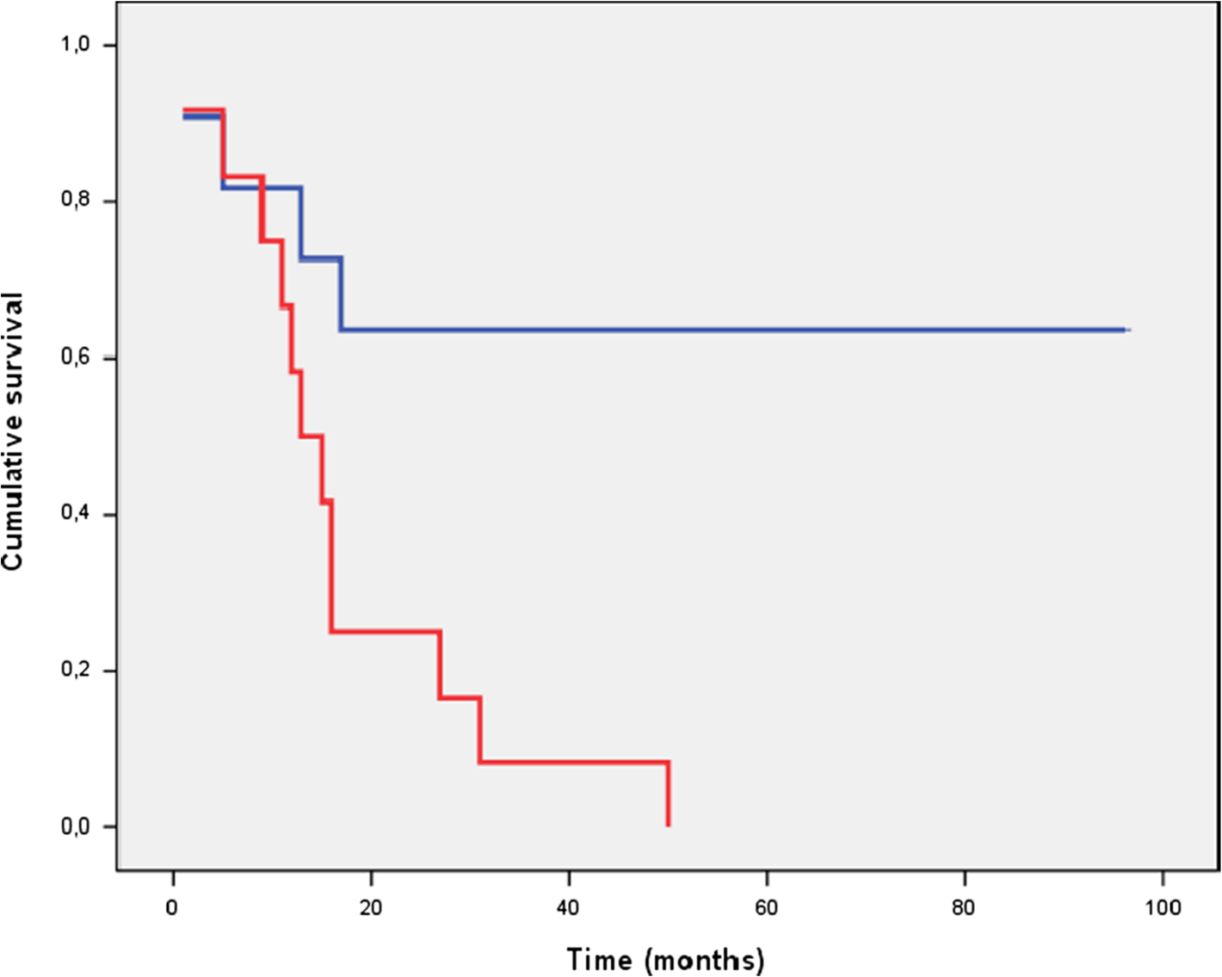
**The disease-free survival (DFS) of patients with advanced-stage tumours (stages III and IV), grouped by apoptotic cell rates.** Patients with high AIs (lower line) present shorter survival durations than patients with low AIs (upper line).

To determine whether AI correlates with prognosis in patients with CRCs, we analysed the association between AI and recurrence-free survival. A significantly lower DFS was demonstrated by patients with tumours that had high AIs than by patients with tumours that had low AIs (p = 0.020). Figure 2 illustrates the Kaplan-Meier curves for recurrence-free survival, comparing high versus low AIs. The DFS at 5 years varied depending on the value of the AI. Thus, for a low AI, DFS at 5 years was 61.8 ± 8.1%, whereas the DFS at 5 years was 28.5 ± 9.6% for a high AI (AI > 1%), with p = 0.020. To determine the independent prognostic value of AI, a multivariate analysis was performed using the Cox proportional hazards model. A high apoptotic rate was significantly associated with an increased recurrence rate, and the independent relative risk (RR) was 2.03 (with a 95% confidence interval (CI) of 1.04-4.14). As expected, an advanced tumour stage was also significantly associated with a poor DFS (RR 2.48; the 95% CI was 1.10-5.59). With respect to the analysis of DFS by stages, we observed that a high AI was associated with a shorter survival in more advanced disease (stages III and IV) (p = 0.004) (Figure 3). This association was not present in earlier stages (data not shown).

### Overall survival (OS)

A low AI was associated with a more favourable OS (OS at 5 years: 74.6 ± 8.8%), whereas a high AI was correlated with a poor outcome (OS at 5 years: 43.7 ± 11.9%, with p = 0.027). In a multivariate analysis, we found that AI and tumour stage were independent prognostic factors for OS, with RR values of 2.18 (with a 95% CI of 1.08-4.37) and 2.41 (with a 95% CI of 1.20-4.85), respectively.

## Discussion

Normal tissue homeostasis is maintained by a balance between the proliferation and apoptosis of colonic epithelial cells. These activities are specifically localised within the intestinal crypt. In normal mucosa, predominantly proliferative activities are found at the lower part of the crypt, where stem cells reside and split into daughter cells; by contrast, greater apoptotic frequencies are localised to the top of the crypt because daughter cells proliferate and differentiate during their migration up and are shed into the lumen or eliminated by apoptosis upon reaching the top of the crypt [11]. These gradients are reversed in adenomas, which feature increased proliferation towards the upper part of the crypt and more elevated levels of apoptosis at the bottom of the crypt; a greater overall rate of apoptosis is found in adenomas than in normal crypts [4,6,12]. By contrast, apoptosis is not specifically localised in carcinomas [6]. An explanation for these distribution patterns is provided by the role of programmed cell death in the control of genetic damage. The withdrawal of cells with DNA mutations through apoptosis, prevents the replication and expansion of these cells. This protective function explains why higher AIs may be found in tumours than in normal tissue, as these elevated AIs may indicate physiological attempts to eliminate the genetic alterations that are frequently found in neoplastic cells. It is true that tumour cells are able to develop mechanisms to evade apoptosis and become immortal. However, if only the neoplastic cells with mutations that inhibit apoptosis will survive and continue proliferating, then apoptosis serves to select the most aggressive cell specimens for tumour formation.

In addition to genetic damage, other factors, such as nutrient, growth factor or oxygen deficiencies, can also stimulate programmed cell death. Indeed, hypoxia is a common feature of most solid tumours because neoplasias that are undergoing rapid proliferation often overwhelm the capability of existing vessels to provide oxygen. Malignant cells need to adapt to their microenvironment, and this confers a more resistant phenotype to these cells, thereby increasing the risk of tumour progression [4,13].

Another link between cell death and carcinogenesis has been suggested. It has been observed that apoptotic cells can have an effect on the tumour microenvironment and the inmune response in the associated stroma, leading to an activation of neoplastic progression [14]. Caspase 3, a marker for apoptosis, has been proposed as the key signal of dying tumour cells to stimulate the growth or surviving cells after radiotherapy [15].

Thus, proliferation and apoptosis are coupled. However, although it is established knowledge that cell proliferation gradually increases with tumour progression, studies trying to clarify whether the same phenomenon occurs for apoptosis have produced dissenting results. It has been argued that these discrepancies could be related to lack of uniformity in the selection and preparation of tissue, influencing pre-analytical variables, especially cold ischemic time and formalin fixation process [16]. Additionally, differences among the methods that are used for the detection of apoptosis could influence the data that are obtained in these studies. Nevertheless, regardless of the apoptotic detection method that is chosen, most authors have demonstrated an increase in the AIs that are observed during the course of the progression from normal mucosa to adenoma to carcinoma and a good correlation has been found when different methods have been compared, like M30 antibody or cleaved caspase 3 [5-8,17].

Moreover, the primary article that reported a progressive decrease in AIs from normal mucosa to carcinoma [3] incorporates certain important methodological limitations, as Koornstra et al. have observed [16]. Our findings clearly confirm that apoptosis upregulation is implicated in the colorectal carcinogenesis process that transforms normal tissue to premalignant and malignant lesions.

This progressive increase in apoptotic rate during the course of tumour development has been observed not only in CRC but also in other cancer types. For example, the AIs are greater in lymph node metastases than in primary breast carcinomas [18], and AIs are also positively correlated with the pathologic grades of gliomas [19]. AI elevation has also been observed in the carcinogenesis of the endometrium [20] or the lung [21]; according to the observations of Törmänen et al., the demonstrated AI in these contexts also increased with the severity of the dysplasias that were observed. In experiments with neoplastic stomach samples, less apoptosis was present in the early stages of gastric cancers than in advanced stages of these cancers, an observation that has been addressed by several different authors [22,23].

Once we have demonstrated the participation of apoptosis in carcinogenesis, the next logical step is to test its association with patient prognoses. These tests have been conducted both in CRC and in non-colorectal neoplasms [24,25]. The existing studies that have attempted to address the prognostic significance of AI in CRC have produced inconclusive results. Several investigations have demonstrated that reduced apoptosis is associated with adverse outcomes and metastatic stages, some of them only in case of distal colon carcinomas [26], but more recent studies have suggested an inverse relationship between AI and survival [9,10,27]. Because proliferation and apoptosis are closely related, it would not be unusual to discover that compared with tumours that develop over a more indolent course, more aggressive tumours are more proliferative and present higher AIs. In fact, both Watanabe et al. [28] and Evertsson et al. [29] observed an increase in apoptosis and proliferation activities during the course of tumour progression from early to advanced stages of CRC. The same conclusion was reached in a study of rectal cancer by Kim et al. [30]; these researchers also linked apoptosis to lymphatic invasion. Recently, authors that were investigating the relationship between KRAS mutations and prognoses for CRC cases have observed that KRAS mutations lead to the higher turnover of colorectal tumour cells, which stimulates both mitosis and apoptosis and is related to a poor survival of CRC [31].

Other markers have been used to study the relationship between apoptosis and survival. Thus, the prognostic significance of cleaved caspase 3 has been evaluated with varying conflicting results [14,32,33]. Alternatively, when apoptosis was measured by M30 antibody, an association between elevated AI and reduced survival was observed [34,35].

In the present study, we report that a high AI is significantly associated with both decreased DFS and reduced OS among patients with invasive CRC. To our knowledge, this is the first time that these data are reported. As we expected, the AI was higher in the more advanced stages of the illness, a result that is in accordance with previously published findings [29,36]. Thus, apoptotic rates increase as tumours progress. Moreover, in advanced disease stages, a high AI is associated with a shorter DFS duration. Therefore, the determination of AI in stages III and IV may help to identify patients who might expect a worse outcome and would therefore most likely benefit from more intense regimens of chemotherapy.

In summary, our study demonstrates an increase in apoptosis during colorectal carcinogenesis and a distinct correlation between apoptotic rates and survival outcomes. At the present time, the importance of apoptosis and antiapoptotic signalling pathways in the pathogenesis and prognosis of CRC is being increasingly recognised. Molecules involved in these pathways represent potential diagnostic markers and therapeutic targets and are therefore the focus of numerous research efforts.

## Conclusions

We conclude that disregulated apoptosis is an important event during CRC development and progression. Our study demonstrates an increase in apoptosis during colorectal carcinogenesis and a distinct correlation between apoptotic rates and survival. Higher AIs are associated with more aggressive tumours and a poorer prognosis for patients with CRC. To our knowledge, this is the first study reporting evidence that high AIs are significantly associated with both decreased DFS and reduced OS among patients with invasive CRC. Therefore, AI is an independent prognostic factor in CRC that could help to guide therapy decisions, and molecules involved in apoptosis regulation represent potential diagnostic markers and therapeutic targets.

## Competing interests

The authors declare that they have no competing interests.

## Authors’ contributions

MR conceived of the study, participated in its design, coordination, analysis and interpretation of data and supervised the writing of the manuscript. JA, TP, IR, and RF participated in data acquisition, quality control, analysis and interpretation. RC and MM performed the statistical analysis. JA drafted the manuscript. EPR contributed in manuscript editing. AR made substantial contributions to study design and manuscript review. All the authors made intellectual contributions, read and approved the final manuscript.

## Pre-publication history

The pre-publication history for this paper can be accessed here:

http://www.biomedcentral.com/1472-6890/13/24/prepub

## References

[B1] Parkin DM Bray F Ferlay J Pisani P Global cancer statistics 2002 CA Cancer J Clin 2005 55 74 108 10.3322/canjclin.55.2.74 15761078

[B2] Fearon ER Vogelstein B A genetic model for colorectal tumorigenesis Cell 1990 61 759 767 10.1016/0092-8674(90)90186-I 2188735

[B3] Bedi A Pasricha PJ Akhtar AJ Barber JP Bedi GC Giardiello FM Zehnbauer BA Hamilton SR Jones RJ Inhibition of apoptosis during development of colorectal cancer Cancer Res 1995 55 1811 1816 7728743

[B4] Aotake T Lu CD Chiba Y Muraoka R Tanigawa N Changes of angiogenesis and tumor cell apoptosis during colorectal carcinogenesis Clin Cancer Res 1999 5 135 142 9918211

[B5] Baretton GB Diebold J Christoforis G Vogt M Müller C Dopfer K Schneiderbanger K Schmidt M Löhrs U Apoptosis and immunohistochemical bcl-2 expression in colorectal adenomas and carcinomas. Aspects of carcinogenesis and prognostic significance Cancer 1996 77 255 264 10.1002/(SICI)1097-0142(19960115)77:2<255::AID-CNCR6>3.0.CO;2-L 8625232

[B6] Sinicrope FA Roddey G McDonnell TJ Shen Y Cleary KR Stephens LC Increased apoptosis accompanies neoplastic development in the human colorectum Clin Cancer Res 1996 2 1999 2006 9816159

[B7] Koornstra JJ Rijcken FE De Jong S Hollema H de Vries EG Kleibeuker JH Assessment of apoptosis by M30 immunoreactivity and the correlation with morphological criteria in normal colorectal mucosa, adenomas and carcinomas Histopathology 2004 44 9 17 10.1111/j.1365-2559.2004.01739.x 14717663

[B8] De Oliveira LF De Oliveira CH Barrezueta LF Fujiyama Oshima CT Silva JA Jr Gomes TS Pinheiro N Jr Neto RA Franco M Immunoexpression of inhibitors of apoptosis proteins and their antagonist SMAC/DIABLO in colorectal carcinoma: correlation with apoptotic index, cellular proliferation and prognosis Oncol Rep 2009 22 295 303 19578769

[B9] Bendardaf R Ristamäki R Kujari H Laine J Lamlum H Collan Y Pyrhönen S Apoptotic index and bcl-2 expression as prognostic factors in colorectal carcinoma Oncology 2003 64 435 442 10.1159/000070304 12759543

[B10] Hilska M Collan YU O Laine VJ Kössi J Hirsimäki P Laato M Roberts PJ The significance of tumor markers for proliferation and apoptosis in predicting survival in colorectal cancer Dis Colon Rectum 2005 48 2197 2208 10.1007/s10350-005-0202-x 16400510

[B11] Barker N van de Wetering M Clevers H The intestinal stem cell Genes Dev 2008 22 1856 1864 10.1101/gad.1674008 18628392 PMC2735277

[B12] Boman BM Huang E Human colon cancer stem cells: a new paradigm in gastrointestinal oncology J Clin Oncol 2008 26 2828 2838 10.1200/JCO.2008.17.6941 18539961

[B13] Rohwer N Cramer T Hypoxia-mediated drug resistance: novel insights on the functional interaction of HIFs and cell death pathways Drug Resist Updat 2011 14 191 201 10.1016/j.drup.2011.03.001 21466972

[B14] Noble P Vyas M Al-Attar A Durrant S Scholefield J Durrant L High levels of cleaved caspase-3 in colorectal tumour stroma predict good survival Br J Cancer 2013 108 2097 2105 10.1038/bjc.2013.166 23591201 PMC3670501

[B15] Huang Q Li F Liu X Li W Shi W Liu FF O'Sullivan B He Z Peng Y Tan AC Zhou L Shen J Han G Wang XJ Thorburn J Thorburn A Jimeno A Raben D Bedford JS Li CY Caspase 3-mediated stimulation of tumor cell repopulation during cancer radiotherapy Nat Med 2011 17 860 866 10.1038/nm.2385 21725296 PMC3132290

[B16] Koornstra JJ de Jong S Hollema H de Vries EG Kleibeuker JH Changes in apoptosis during the development of colorectal cancer: a systematic review of the literature Crit Rev Oncol Hematol 2003 45 37 53 10.1016/S1040-8428(01)00228-1 12482571

[B17] Carr NJ M30 expression demonstrates apoptotic cells, correlates with in situ end-labeling, and is associated with Ki-67 expression in large intestinal neoplasms Arch Pathol Lab Med 2000 124 1768 1772 11100055 10.5858/2000-124-1768-MEDACC

[B18] Villar E Redondo M Rodrigo I García J Avila E Matilla A bcl-2 Expression and apoptosis in primary and metastatic breast carcinomas Tumour Biol 2001 22 137 145 10.1159/000050608 11275791

[B19] Zhen HN Zhang X Hu PZ Yang TT Fei Z Zhang JN Fu LA He XS Ma FC Wang XL Survivin expression and its relation with proliferation, apoptosis, and angiogenesis in brain gliomas Cancer 2005 104 2775 2783 10.1002/cncr.21490 16284993

[B20] Atasoy P Bozdoğan O Erekul S Bozdoğan N Bayram M Fas-mediated pathway and apoptosis in normal, hyperplastic, and neoplastic endometrium Gynecol Oncol 2003 91 309 317 10.1016/S0090-8258(03)00411-6 14599860

[B21] Törmänen U Nuorva K Soini Y Pääkkö P Apoptotic activity is increased in parallel with the metaplasia-dysplasia-carcinoma sequence of the bronchial epithelium Br J Cancer 1999 79 996 1002 10.1038/sj.bjc.6690159 10070903 PMC2362669

[B22] Koshida Y Saegusa M Okayasu I Apoptotosis, cell proliferation and expression of Bcl-2 and Bax in gastric carcinomas: immunohistochemical and clinicopathological study Br J Cancer 1997 75 367 373 10.1038/bjc.1997.60 9020481 PMC2063381

[B23] Ishii HH Gobé GC Pan W Yoneyama J Ebihara Y Apoptosis and cell proliferation in the development of gastric carcinomas: associations with c-myc and p53 protein expression J Gastroenterol Hepatol 2002 17 966 972 10.1046/j.1440-1746.2002.02805.x 12167117

[B24] Törmänen U Eerola AK Rainio P Vähäkangas K Soini Y Sormunen R Bloigu R Lehto VP Pääkkö P Enhanced apoptosis predicts shortened survival in non-small cell lung carcinoma Cancer Res 1995 55 5595 5602 7585640

[B25] De Jong JS van Diest PJ Baak JP Number of apoptotic cells as a prognostic marker in invasive breast cancer Br J Cancer 2000 82 368 373 10646890 10.1054/bjoc.1999.0928 PMC2363300

[B26] Sinicrope FA Hart J Hsu HA Lemoine M Michelassi F Stephens LC Apoptotic and mitotic indices predict survival rates in lymph node-negative colon carcinomas Clin Cancer Res 1999 5 1793 1804 10430084

[B27] Garrity MM Burgart LJ Mahoney MR Windschitl HE Salim M Wiesenfeld M Krook JE Michalak JC Goldberg RM O'Connell MJ Furth AF Sargent DJ Murphy LM Hill E Riehle DL Meyers CH Witzig TE North Central Cancer Treatment Group Prognostic value of proliferation, apoptosis, defective DNA mismatch repair, and p53 overexpression in patients with resected Dukes' B2 or C colon cancer: a north central cancer treatment group study J Clin Oncol 2004 22 1572 1582 10.1200/JCO.2004.10.042 15117979

[B28] Watanabe I Toyoda M Okuda J Tenjo T Tanaka K Yamamoto T Kawasaki H Sugiyama T Kawarada Y Tanigawa N Detection of apoptotic cells in human colorectal cancer by two different in situ methods: antibody against single-stranded DNA and terminal deoxynucleotidyl transferase-mediated dUTP-biotin nick end-labeling (TUNEL) methods Jpn J Cancer Res 1999 90 188 193 10.1111/j.1349-7006.1999.tb00732.x 10189889 PMC5926049

[B29] Evertsson S Bartik Z Zhang H Jansson A Sun XF Apoptosis in relation to proliferating cell nuclear antigen and Dukes' stage in colorectal adenocarcinoma Int J Oncol 1999 15 53 58 10375593 10.3892/ijo.15.1.53

[B30] Kim YH Lee JH Chun H Nam SJ Lee WY Song SY Kwon OJ Hyun JG Sung IK Son HJ Rhee PL Kim JJ Paik SW Rhee JC Choi KW Apoptosis and its correlation with proliferative activity in rectal cancer J Surg Oncol 2002 79 236 242 10.1002/jso.10063 11920781

[B31] Liu X Jakubowski M Hunt JL KRAS gene mutation in colorectal cancer is correlated with increased proliferation and spontaneous apoptosis Am J Clin Pathol 2011 135 245 252 10.1309/AJCP7FO2VAXIVSTP 21228365

[B32] Leonardos L Butler LM Hewett PJ Zalewski PD Cowled PA The activity of caspase-3-like proteases is elevated during the development of colorectal carcinoma Cancer Lett 1999 143 29 35 10.1016/S0304-3835(99)00176-7 10465334

[B33] Jonges LE Nagelkerke JF Ensink NG van der Velde EA Tollenaar RA Fleuren GJ van de Velde CJ Morreau H Kuppen PJ Caspase-3 activity as a prognostic factor in colorectal carcinoma Lab Invest 2001 81 681 688 10.1038/labinvest.3780277 11351040

[B34] Evans C Morrison I Heriot AG Bartlett JB Finlayson C Dalgleish AG Kumar D The correlation between colorectal cancer rates of proliferation and apoptosis and systemic cytokine levels; plus their influence upon survival Br J Cancer 2006 94 1412 1419 10.1038/sj.bjc.6603104 16641913 PMC2361288

[B35] Rupa JD de Bruïne AP Gerbers AJ Leers MP Nap M Kessels AG Schutte B Arends JW Simultaneous detection of apoptosis and proliferation in colorectal carcinoma by multiparameter flow cytometry allows separation of high and low-turnover tumors with distinct clinical outcome Cancer 2003 97 2404 2411 10.1002/cncr.11366 12733138

[B36] Elkablawy MA Maxwell P Williamson K Anderson N Hamilton PW Apoptosis and cell-cycle regulatory proteins in colorectal carcinoma: relationship to tumour stage and patient survival J Pathol 2001 194 436 443 10.1002/path.894 11523051

